# Circulating microRNAs and association with methacholine PC_20_ in the Childhood Asthma Management Program (CAMP) cohort

**DOI:** 10.1371/journal.pone.0180329

**Published:** 2017-07-27

**Authors:** Joshua S. Davis, Maoyun Sun, Alvin T. Kho, Kip G. Moore, Jody M. Sylvia, Scott T. Weiss, Quan Lu, Kelan G. Tantisira

**Affiliations:** 1 Channing Division of Network Medicine, Brigham and Women’s Hospital and Harvard Medical School, Boston, Massachusetts, United States of America; 2 Molecular and Integrative Physiological Sciences Program, Department of Environmental Health, Harvard T.H. Chan School of Public Health, Boston, Massachusetts, United States of America; 3 Computational Health Informatics Program, Boston Children’s Hospital, Boston, Massachusetts, United States of America; Universite de Bretagne Occidentale, FRANCE

## Abstract

**Introduction:**

Circulating microRNAs (miRNA) are promising biomarkers for human diseases. Our study hypothesizes that circulating miRNA would reveal candidate biomarkers related to airway hyperresponsiveness (AHR) and provide biologic insights into asthma epigenetic influences.

**Methods:**

Serum samples obtained at randomization for 160 children in the Childhood Asthma Management Program were profiled using a TaqMan miRNA array set. The association of the isolated miRNA with methacholine PC_20_ was assessed. Network and pathway analyses were performed. Functional validation of two significant miRNAs was performed in human airway smooth muscle cells (HASMs).

**Results:**

Of 155 well-detected circulating miRNAs, eight were significantly associated with PC_20_ with the strongest association with miR-296-5p. Pathway analysis revealed miR-16-5p as a network hub, and involvement of multiple miRNAs interacting with genes in the FoxO and Hippo signaling pathways by KEGG analysis. Functional validation of two miRNA in HASM showed effects on cell growth and diameter.

**Conclusion:**

Reduced circulatory miRNA expression at baseline is associated with an increase in PC_20_. These miRNA provide biologic insights into, and may serve as biomarkers of, asthma severity. miR-16-5p and -30d-5p regulate airway smooth muscle phenotypes critically involved in asthma pathogenesis, supporting a mechanistic link to these findings. Functional ASM phenotypes may be directly relevant to AHR.

## I. Introduction

Asthma is a chronic inflammatory respiratory disease that affects greater than 300 million people worldwide [1]. It is characterized by airway obstruction due to a combination of smooth muscle hyperresponsiveness and inflammation [2]. The economic costs for asthma including drug therapy and hospitalizations is significant [3]. It remains challenging to generate risk assessment, predict prognosis, and determine optimal treatment response in asthmatics.

Circulating microRNAs (miRNAs) are promising biomarkers for human diseases [4] and may be helpful in a variety of clinical scenarios from risk assessment to monitoring response to treatment [5]. miRNA characteristics and function have been well described in the literature [6]. In brief, miRNAs are a class of small RNAs that inhibit gene expression by binding to the 3’-untranslated region (UTR) of messenger RNAs to degrade or suppress the translation of the mRNA. Given the availability of miRNA mimics and antagonists, these small RNAs have been proposed as therapeutic targets. Circulating miRNAs are highly stable in the serum [7]. miRNA plasma biomarkers have been proposed for neurological conditions [8], cancer detection/prognosis [9], cardiovascular disease [10], and other conditions including an emerging role in respiratory diseases [11]. Translational methods have been applied in order to generate screening tests [12].

Prior studies of circulating miRNA in asthma have been performed. One study explored serum miRNA expression and detected three miRNAs in childhood asthma patients with significantly higher expression than healthy controls [13]. Other studies have shown differential expression of miRNA in epithelial and airway cells between asthma and healthy controls [14]. A recent study explored differential expression of circulating miRNA in asthmatics, nonasthmatic patients with allergic rhinitis, and normal patients and was able to identify a subset of circulating miRNA expressed in asthmatic and allergic rhinitis patients [15]. Studies are lacking regarding quantitative severity measures, which may be more revealing of specific asthma pathobiology and resistant to misclassification bias.

Methacholine PC_20_ is a quantitative marker of airways responsiveness, which is a cardinal feature of asthma and has been tightly linked to exacerbations and other asthma outcomes. Our study investigated the association of circulating miRNA with methacholine PC_20_ at time of randomization in the Childhood Asthma Management Program (CAMP) [16]. Airway hyperresponsiveness (AHR) in CAMP was an inclusion criterion for the trial; the degree of airway responsiveness has been linked to disease severity [17]. Our hypothesis is that specific miRNAs may be mediating AHR thereby providing unique biologic insights into asthma pathogenesis. We detected AHR related miRNAs previously associated with asthma, but not PC_20_, in addition to a novel association of miR-296, that may have an immunomodulatory effect. Pathway analysis of the PC_20_ associated miRNAs resulted in identification of two pathways known to be biologically significant for AHR. Functional validation of miR-16-5p and miR-30d-5p in human airway smooth muscle cells (HASM) demonstrated effects on cell growth and average cell diameter, respectively, supporting a mechanistic link to these findings.

## II. Materials and methods

CAMP (Clinicaltrials.gov register: NCT00000575) was a multi-center, randomized, double-blinded clinical trial evaluating safety and efficacy of inhaled budesonide vs. nedocromil vs. placebo in 1041 pediatric patients over a mean 4.3 years. Trial design and methodology have been detailed [18]. Inclusion criteria were notable for children aged 5–12 years, chronic asthma symptoms, and PC_20_ < 12.5 mg/mL. Children were excluded if their asthma was severe, for a confounding or complicating condition, or if the child could not perform acceptable spirometry or methacholine challenge. Methacholine challenge was performed 2 weeks prior to randomization [16].

Blood serum samples from 160 CAMP subjects obtained at randomization were profiled for miRNA as described [19]. Technical replicates were assessed in ~10% of the population cohort demonstrating high miRNA-miRNA correlations. To limit the effect of race on miRNA expression (20), all subjects were self-identified non-Hispanic Caucasians. miRNA were annotated with usage of miRBase [20] release 21 (www.mirbase.org/). Analysis was limited to miRNAs detected in ≥50% of samples. The CAMP Genetics Ancillary Study was approved by each individual study center’s Internal Review Board (IRB). Informed consent and assent was obtained from parents and participants, respectively.

For data analysis, quantile normalization on the detected miRNAs was performed sample-wise to the mean of the data matrix using MatLab (MathWorks Inc., Natick, MA) function *quantilenorm*. Least squares linear regression (both univariate and multivariate) was performed using R [21] to identify miRNA (miR cycle threshold or CT value) associated with the pulmonary function phenotype of interest, log_2_ PC_20_. A least squares multivariate linear regression model including miR CT value, age, sex, and height was also calculated for each miRNA. A sensitivity analysis to assess outlier influence, and non-parametric models was also performed. The p-values were corrected using the Benjamini and Hochberg false discovery rate (FDR).

The miRNA dataset is available at the NCBI Gene Expression Omnibus (GEO, http://www.ncbi.nih.gov/geo/) GSE74770.

A regulatory network between miRNA and genes was created with usage of Cytoscape (http://www.cytoscape.org/) [22] and CyTargetLinker (http://projects.bigcat.unimaas.nl/cytargetlinker/) [23] with Regulatory Interaction in Network Analysis (RegIN) miRTarBase release 6.1 (http://projects.bigcat.unimaas.nl/cytargetlinker/regins/regins-mirtarbase/) [24]. The Database for Annotation, Visualization and Integrated Discovery (DAVID, Version 6.8 (10/2016), https://david.ncifcrf.gov/home.jsp [25] was used for KEGG [26, 27] pathway analysis and gene ontology.

Functional validation of two significant miRNA was performed in human airway smooth muscle (HASM) cells as previously detailed [28]. The cells were transfected with 10nM of either scramble control (AllStars Negative Control siRNA, Qiagen) or miR mimic (Qiagen) using RNAiMax (Life Technology) according to manufacturer’s protocol. Seventy-two hours after transfection, cells were trypsinized for 8 minutes and then measured for both cell number and cell size by Moxi Z Cell Analyzer (Orflo). Cell growth was presented as the percentage of cell number relative to scramble control. Average cell diameter (um) was compared in mimic-transfected versus scramble-transfected HASM cells. Data (mean±SE) were obtained from three independent experiments.

## III. Results

### Study population

Population characteristics of the 160 CAMP subjects are shown in Table 1. The cohort was limited to self-identified non-Hispanic whites due to the significant effects of race on miRNA expression [29]. For the selected individuals, the global characteristics at randomization are representative of the larger CAMP non-Hispanic white cohort.

**Table 1:** Characteristics of the CAMP cohort subset.

Characteristic	Value (Standard Deviation)
Age - yr	8.8 (2.1)
Sex - no. (%)	Female - 73 (45.6%), Male - 87 (54.4%)
Height - cm	132.7 (13.6)
PC_20_ - mg/mL	1.95 (2.38)
log_2_(PC_20_) - mg/mL	0.06 (1.66)

### Circulatory miRNA association with PC_20_

**Fig 1:**
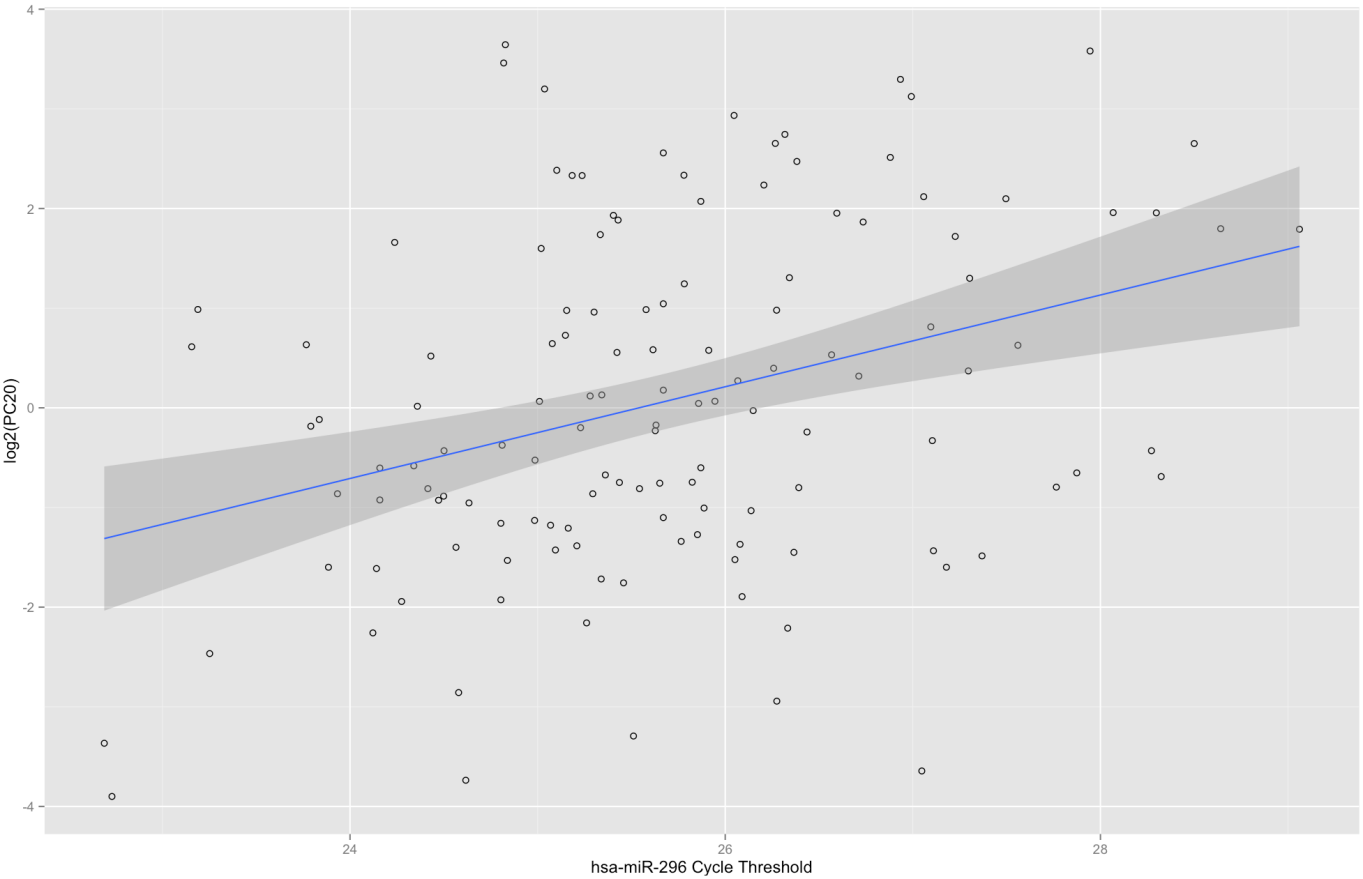
Representative scatter plot of serum miR-296 cycle threshold and log_2_PC_20_ in the CAMP cohort with least squares regression line and 95% confidence interval.

There were a total of 754 non-housekeeping miRNA mapping to mirBase release 21 on the array, and 155 (20.6%) miRNA were detected in at least 50% of the samples. Eight microRNAs were significantly associated with PC_20_ (Table 2), based on a nominal p-value < 0.05 at a FDR p-value < 0.20. The latter was chosen as a higher cut-off given the nature of this hypothesis generating experiment. Based on prior literature, five of these eight miRNA (63%) had prior evidence of differential expression in human asthma. All associations had a positive slope such that as miR cycle threshold increased so did the PC_20_; this corresponds to a relationship of increasing miR CT (decreasing miRNA expression) with increasing PC_20_ (decreasing AHR). The strongest association was found with PC_20_ and hsa-miR-296-5p, as shown in Fig 1. Sensitivity analysis (S1 Table) revealed no significant changes in parameters for the models with the exception of non-significance of hsa-miR-30d. Subsequent multivariate analysis including miR CT, age, sex, and height was consistent with the univariate model (S2 Table). Nonparametric analysis including both rank-order univariate and multivariate models were also performed and were consistent with the parametric models except for the significance of hsa-miR-451a in the nonparametric model (S3 and S4 Tables). Further investigation of miR-30d demonstrated significance in the parametric and non-parametric models with miR-30d cycle threshold characterized by principally having high and low CT values (bimodality) rather than unimodality. This bimodality likely explains non-significance in the sensitivity analysis, while suggesting that miR-30d may still have functional relationship with AHR.

**Table 2:** Circulatory miRNA association by least squares linear regression with methacholine PC_20_ (univariate model, unranked) with detection of miRNA in at least 50% of samples.

miR	Asthma Associated?	miR slope	miR p-value	95% CI Lower	95% CI Upper
hsa-miR-296-5p	N	0.460	0.0001*	0.238	0.683
hsa-miR-548b-5p	N	0.328	0.002*	0.126	0.531
hsa-miR-138-5p	Y	0.368	0.003*	0.129	0.608
hsa-miR-16-5p	Y	0.197	0.005*	0.061	0.332
hsa-miR-1227-3p	N	0.327	0.005*	0.100	0.555
hsa-miR-30d-5p	Y	0.201	0.006*	0.060	0.342
hsa-miR-203a-3p	Y	0.203	0.007*	0.057	0.350
hsa-miR-128-3p	Y	0.587	0.012*	0.132	1.042
hsa-miR-942-5p	N	0.242	0.015	0.047	0.436
hsa-miR-451a	N	0.197	0.016	0.037	0.357
hsa-miR-212-3p	N	0.290	0.020	0.046	0.533
hsa-miR-143-3p	N	0.387	0.035	0.028	0.747
hsa-miR-638	Y	0.208	0.048	0.002	0.414
hsa-miR-25-3p	N	0.219	0.049	0.001	0.437

* Significant by FDR adjusted p-value, p < 0.20 cut-off

### Pathway and ontology analysis

**Fig 2:**
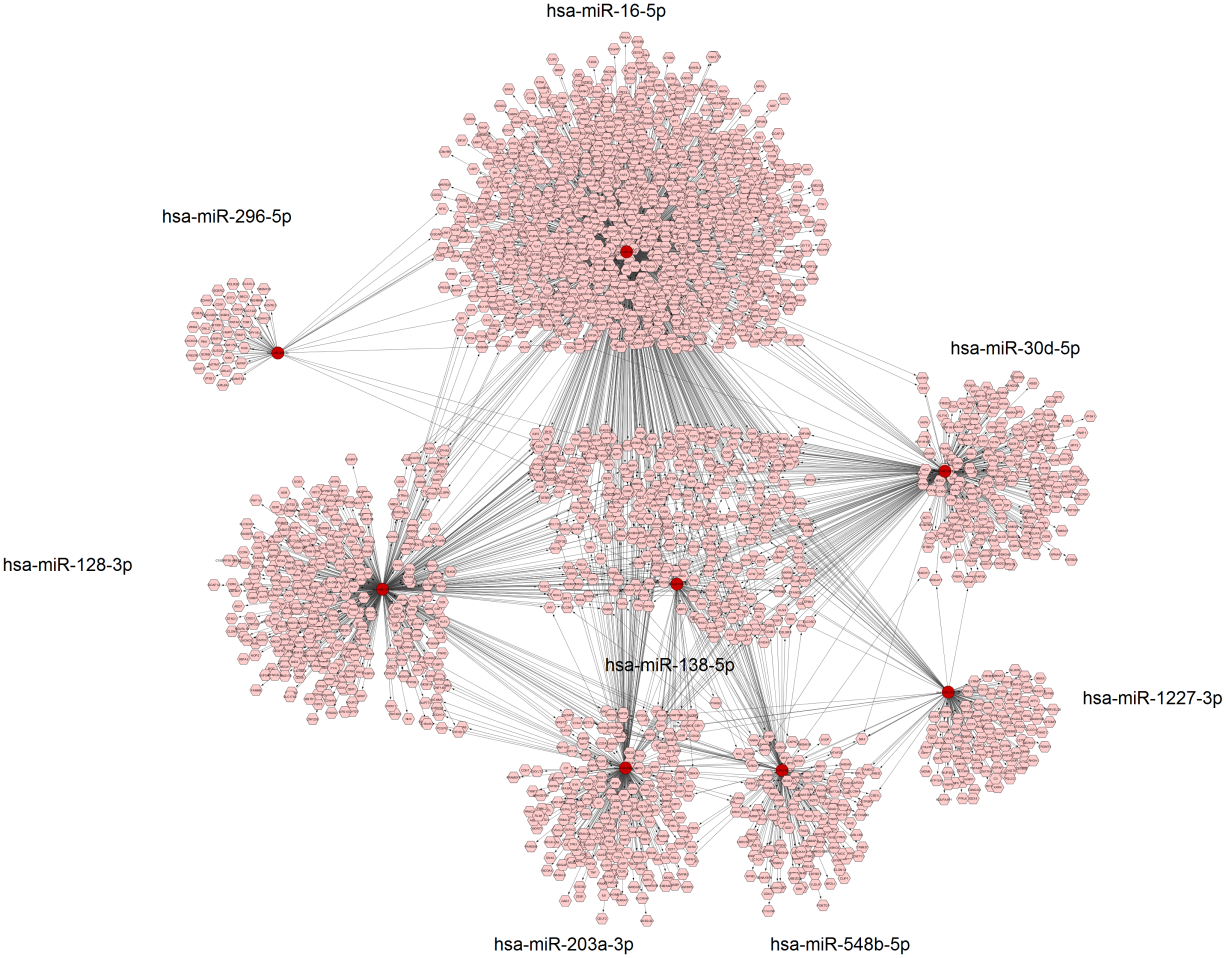
miRNA (red circle) and validated miRNA targeted genes (light magenta circles) predicted by miRTarbase 6.1 in Cytoscape CyTargetLinker with miR-16 having a central connection to other miRNA in the gene network.

**Fig 3:**
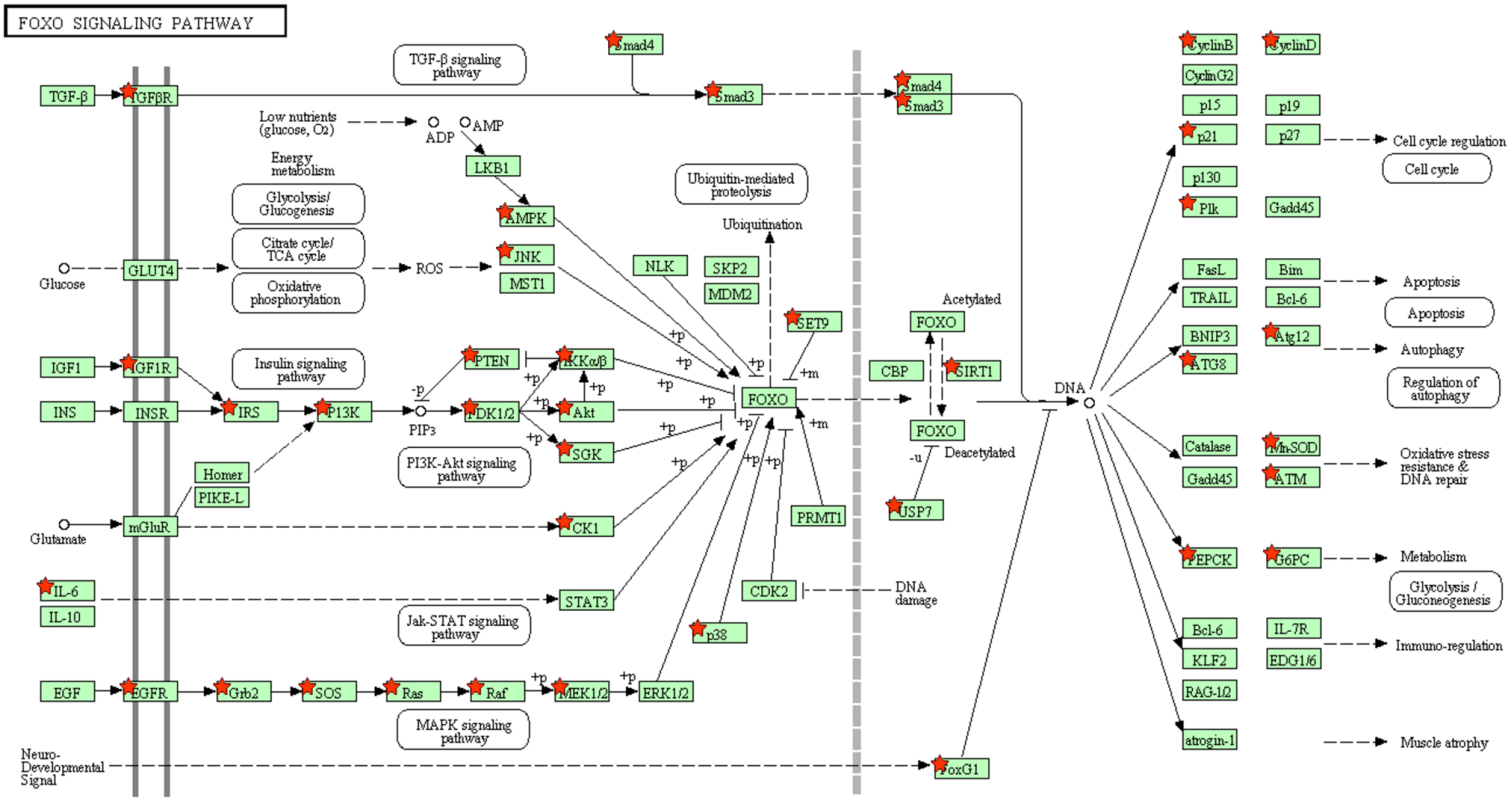
DAVID KEGG pathway analysis; miR targeted genes (red star) are involved in the FoxO Signaling Pathway.

**Fig 4:**
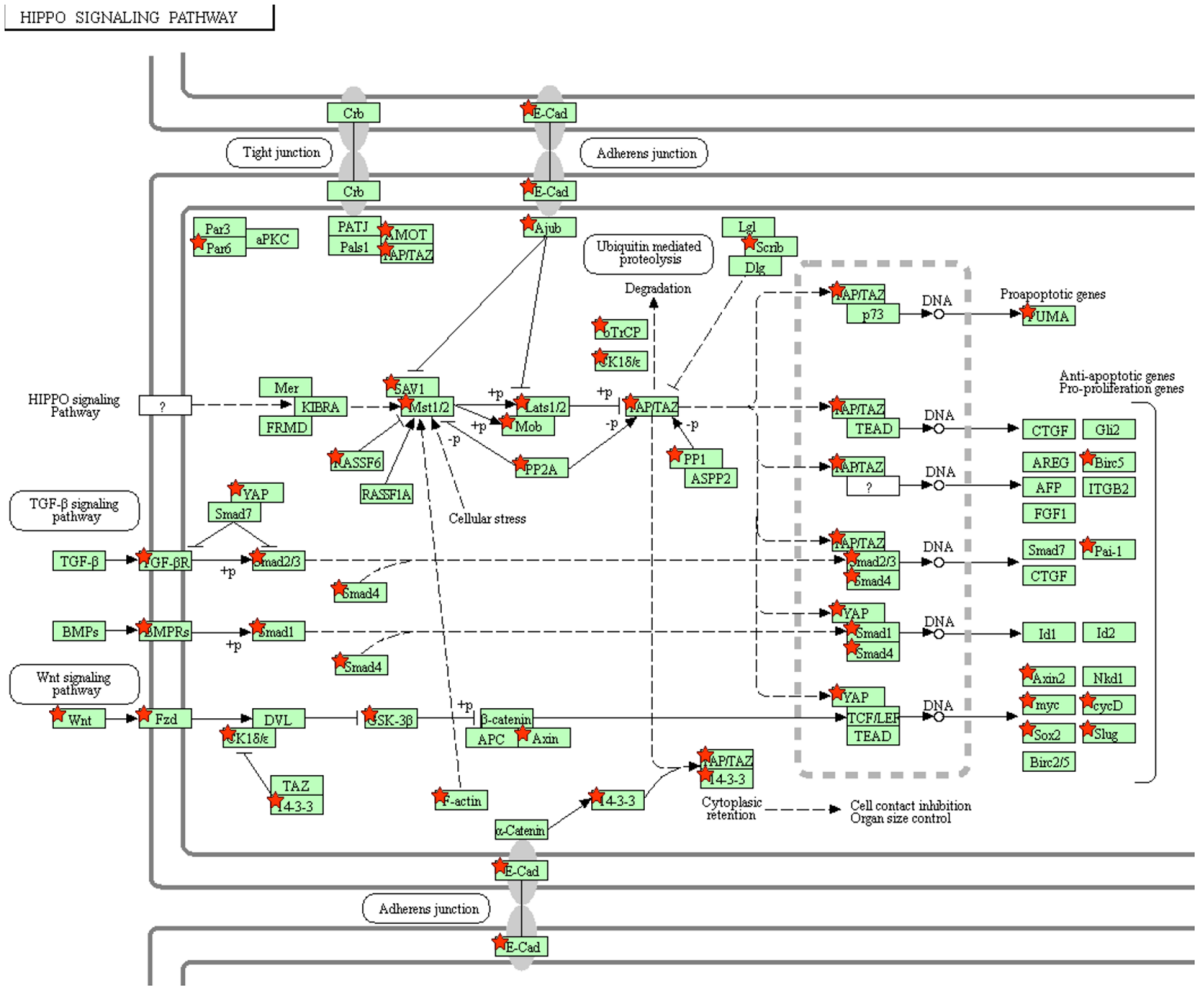
DAVID KEGG pathway analysis; miR targeted genes (red star) are involved in the Hippo Signaling Pathway.

Pathway analysis of the significant miRNAs (S5 Table) was performed with usage of Cytoscape and CyTargetLinker. The miRNA based on both nominal and FDR p-values were used to generate and create a network with Cytoscape and CyTargetLinker (Fig 2) containing multiple genes. The resultant genes were analyzed with DAVID for KEGG (Kyoto Encyclopedia of Genes and Genomes) pathway analysis with the FoxO and Hippo signaling pathways being the most relevant to asthma (Table 3, Figs 3 and 4).

**Table 3:** DAVID Top 10 KEGG pathway analysis of genes directed from validated miRNA targeting.

Term	Number of Genes in Pathway	Percent of Genes Compared to Total (%)	P-value	Corrected P-value (Benjamini)
Signaling pathways regulating pluripotency of stem cells	53	2.0	9.0 x 10^−10^	2.6 x 10^−7^
Pathways in cancer	103	3.9	1.7 x 10^−7^	2.5 x 10^−5^
Pancreatic cancer	27	1.0	3.0 x 10^−6^	1.1 x 10^−4^
**FoxO signaling pathway**	**44**	**1.7**	**2.8 x10**^**-6**^	**1.1 x 10**^**−4**^
**Hippo signaling pathway**	**48**	**1.8**	**2.5 x 10**^**−6**^	**1.2 x 10**^**−4**^

Notes: Threshold for count of 2, EASE 0.1. Table sorted by corrected P-value (Benjamini). The total number of genes with DAVID ID is 2665.

Gene ontology (GOTERM_BP_DIRECT) analysis also revealed functionality of the network related to translation, RNA processing, post-transcriptional regulation of gene expression, ncRNA metabolic process, and other processes (S6 Table). These functions are consistent with the known actions of miRNA targeting.

### Functional validation

**Fig 5:**
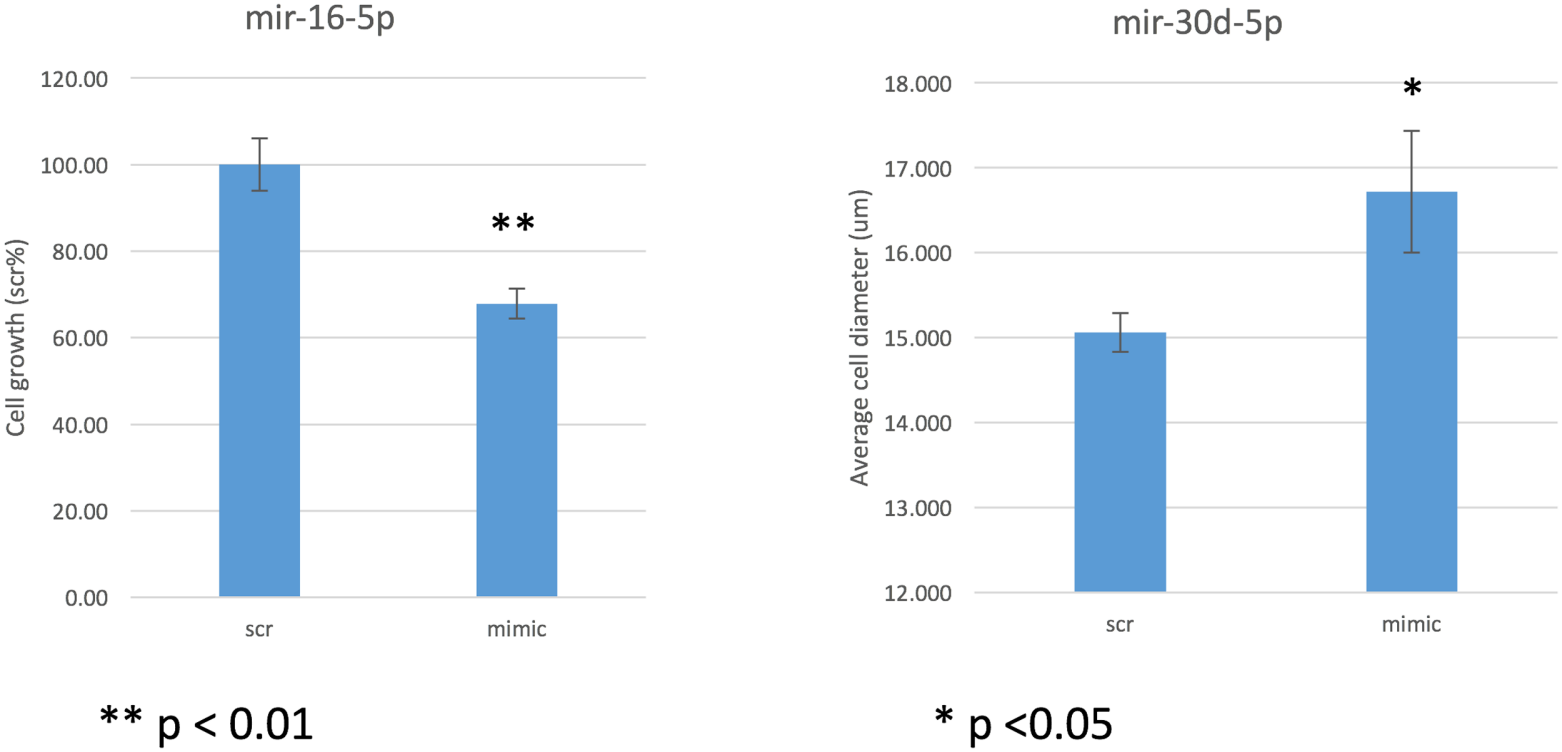
Effect of miR-16-5p and miR-30d-5p on cell growth and average cell diameter, respectively compared to scramble control. HASM cells were transfected with 10 nM of either scramble control or miR-16-5p mimic (left panel; or miR-30d-5p in right panel). Seventy-two hours after transfection, cells were trypsinized and measured for both cell number and cell size by Moxi Z Cell Analyzer. Cell growth was presented as the percentage of cell number relative to the scramble control. Average cell size (um in diameter) was compared in mimic-transfected versus scramble-transfected HASM cells. Data were obtained from three independent experiments. For the miR-16 and miR-30d experiments, the p-value is 0.0009 and 0.03, respectively.

Based on our prior miRNA sequencing of human airway smooth muscle cells, [28] of the miRNAs in the PC_20_ network (Fig 2), two, miR-16-5p and miR-30d-5p, are significantly expressed. We therefore evaluated the effect of these miRNA on HASM phenotypes using miR-mimics. Mimics of miR-16-5p decreased and miR-30d-5p increased cell growth and average cell diameter, respectively, compared to scramble control (Fig 5).

## IV. Discussion

In this study, we examined serum samples from 160 CAMP asthmatics and found 8 miRNA significantly associated with PC_20_, a defining measure of airways hyperresponsiveness. Based on prior literature, five of the eight miRNA (63%) had evidence of differential expression related to human asthma, but not PC_20_, with a good portion of these in case-control studies of human bronchial epithelial cells. Three novel miRNAs were identified, including our strongest association, miR-296-5p. Pathway analysis of the miRNA targets implicates effects of both the Hippo and FoxO signaling pathways with both pathways implicated in airways hyperresponsiveness [30, 31]. Lastly, functional validation demonstrated that miR-16-5p resulted in decreased airway smooth muscle cell growth and miR-30d-5p increased airway smooth muscle cell size compared to scramble controls.

Our most significant association was found with hsa-miR-296-5p (Table 2). There are no previous reports in the literature regarding this miRNA in association with asthma. miR-296 targets *IKBKE*, which is involved in signaling pathways including Toll-like receptor signaling and signal transduction prompting apoptosis [32]. *IKBKE* is highly expressed in immune cells and is a known target of the NFκB gene [33]. The NFκB pathway’s involvement in asthma and inflammation has been well described in the literature [34], and includes modulation of AHR in allergen challenged mice [35]. Moreover, *IKBKE* itself is a known therapeutic target for asthma, with IKBKE targeting demonstrating significant attenuation of airways responsiveness and inflammation in a murine model of asthma [36]. Therefore, miR-296 may attenuate immune response and could modulate AHR via the NFκB pathway.

miR-16-5p was also significant in our study and differential expression of this miRNA in asthmatic airway cells has been reported [37]. Expression profiling of human airway biopsies has showed miR-16 to be highly expressed, leading to the hypothesis that miR-16 along with other miRNAs may have a significant influence on gene expression in the airways [38]. Our network analysis demonstrated that miR-16 plays a key role as the central hub, both interacting with other miRNAs and mediating expression of dozens of genes (Fig 2). Thus, miR-16 appears to play a notable role in the modulation of genes influencing airways hyperresponsiveness in asthma. In addition to its central effect on downstream gene expression, miR-16 mimics result in decreased airway smooth muscle growth. While the exact significance of this finding is unknown, prior work focused on small airway cell layers suggests that differential growth between layers may mediate different effects on airway buckling [39].

As mentioned, several of our other AHR associated miRNA, including hsa-miR-30d, -128, -138, and -203a, have been detected in studies involving human airway cells of asthmatics [14]. The association of hsa-miR-203 has been validated in epithelial cells from a small number of asthmatics and healthy subjects with identification of the top-ranked predicted target, aquaporin gene (AQp4). In turn, the expression of AQp4 was subsequently noted to be significantly higher in asthmatic cells [14]. Other studies have shown up-regulation of miR-203 in serum of children with atopic dermatitis and increased IgE level [40] in addition to airway epithelial cell apoptosis [41]. Thus miR-203 may indirectly affect airways responsiveness via an inflammatory mechanism. In contrast, our work demonstrates that miR-30d-5p increases average HASM cell diameter compared to scramble controls. Increased airway smooth muscle cell size can result in both further mechanical airway narrowing in addition to increased contribution of inflammatory mediators [42]. Increase in airway smooth muscle tissue mass related to both hypertrophy and hyperplasia has been noted a major driver of airway narrowing and thus AHR in asthmatics [43]. It is very likely that miRNA act via increases in ASM cell size/diameter and thus, mechanistically may directly cause increased AHR (decreased PC20).

Focusing on validated miRNA targets, pathway analysis from our associated miRNAs was notable for multiple genes in both the FoxO and Hippo signaling pathways (Figs 3 and 4). For the former pathway, a mouse experiment showed alternative activation of alveolar macrophages with resultant type 2 allergic airway inflammation with subsequent airway remodeling [30]. For the latter pathway, it has been shown that it is a notable regulatory pathway with versatile function including a key gene (Yes-associated protein or YAP) implicated in airway smooth muscle hyperplasia [31]. Both of these pathways have a plausible link to the phenotype of airways hyperresponsiveness. As noted above, miR-16 also appears to be a central hub in our serum microRNA network and may work in concert with other miRNA to modulate immune pathways and subsequently AHR. Functional validation would be needed for further elucidation of possible molecular mechanisms between miRNAs and asthma related to this pathway. Lastly, gene ontology analysis (S6 Table) demonstrated processes such as RNA processing, post-transcriptional regulation of gene expression, and other likely putative effects of miRNAs.

This study has several strengths including a large sample size of pediatric asthma patients from the CAMP cohort, a large number of interrogated miRNAs, validation of prior associations in the literature with our reported miRNA findings, and subsequent functional validation of miRNA in HASM. The large sample size and number of interrogated miRNAs provides a good breadth of characterization and power to detect associations in light of lower starting concentrations of miRNA in the circulation. Additionally, the CAMP cohort was clinically well characterized with standard methodologies including methacholine challenge testing, which should minimize potential for measurement error. Analysis of biological replicates as discussed in the methods section also showed high miRNA-miRNA correlations. Although the CAMP serums were stored for years prior to this interrogation, prior studies have shown the stored samples can result in reliable miRNA concentrations months to years later [44]. Lastly, miRNA targeting is an imprecise science with new associations being discovered on a regular basis. However, our study used miRTarBase (validated miRNA-target interaction), which assesses only functionally annotated miRNAs, lending functional credence to our network and pathway analyses; this was enhanced by our functional studies in HASM cells.

In summary, this study detected eight circulating miRNAs associated with PC_20_ in a pediatric asthma population with mild-moderate persistent asthma. These miRNA appear to be associated with individual and pathway evidence of immune modulation that could affect AHR; complementary functional validation of miR-16-5p and miR-30d-5p in HASM demonstrate effects on cell growth and diameter, respectively. The majority of these miRNAs had been associated with asthma in prior studies. Nonetheless, the most significant association was a novel association with miR-296, and this miRNA may be a viable serum biomarker for altered immunity and AHR in pediatric asthmatic patients.

Further study of our PC_20_ associated miRNAs, both in terms of external validation and additional functional mechanisms, may provide insight into epigenetic influences in asthma pathobiology and have clinical implications such as risk assessment and treatment responses. Given that miRNA can therapeutically decrease airways responsiveness in murine models of asthma [45–47], future work may also yield novel therapeutic approaches to targeting asthma via miRNA modulation of AHR.

## Supporting information

S1 Table Sensitivity analysis for circulatory miRNA association by least squares linear regression with methacholine PC_20_ (univariate model, unranked) with outlier values removed and with detection of miRNA in at least 50% of samples. (DOCX)

S2 Table Circulatory miRNA association by least squares linear regression with methacholine PC20 (multivariate model adjusting for age, sex, and height, unranked) with detection of miRNA in at least 50% of samples. (DOCX)

S3 Table Circulatory miRNA association by least squares linear regression with methacholine PC20 (univariate model, ranked) with detection of miRNA in at least 50% of samples. (DOCX)

S4 Table Circulatory miRNA association by least squares linear regression with methacholine PC20 (multivariate model adjusting for age, sex, and height, ranked) with detection of miRNA in at least 50% of samples. (DOCX)

S5 Table miRBase Accession numbers for cytoscape (univariate model, unranked). (DOCX)

S6 Table DAVID gene ontology (GO) analysis (GOTERM_BP_DIRECT). (DOCX)
