# Supplementary material for: Circulating microRNAs and association with methacholine PC20 in the Childhood Asthma Management Program (CAMP) cohort
Source: PLoS One. 2017 Jul 27;12(7):e0180329. doi: 10.1371/journal.pone.0180329 (PMC5531511; doi:10.1371/journal.pone.0180329)
Supplement: S1 Table — (DOCX) [file pone.0180329.s001.docx]

**S1 Table: Sensitivity Analysis for Circulatory miRNA Association by Least Squares Linear Regression with methacholine PC_20_ (univariate model, unranked) with outlier values removed and with detection of miRNA in at least 50 % of samples**

| **miR** | **slope** | **p-value** | **FDR p-value** | **95 % CI**  **Lower** | **95 % CI**  **Upper** |
| --- | --- | --- | --- | --- | --- |
| hsa-miR-296-5p | 0.342 | 0.008 | 0.011 | 0.090 | 0.593 |
| hsa-miR-548b-5p | 0.353 | 0.003 | 0.069 | 0.127 | 0.578 |
| hsa-miR-138-5p | 0.361 | 0.005 | 0.090 | 0.109 | 0.613 |
| hsa-miR-16-5p | 0.238 | 0.010 | 0.109 | 0.059 | 0.417 |
| hsa-miR-1227-3p | 0.287 | 0.049 | 0.109 | 0.001 | 0.573 |
| hsa-miR-30d-5p | 0.202 | 0.084 | 0.109 | -0.027 | 0.431 |
| hsa-miR-203a-3p | 0.042 | 0.042 | 0.120 | 0.008 | 0.388 |
| hsa-miR-128-3p | 0.642 | 0.009 | 0.187 | 0.162 | 1.123 |
| hsa-miR-942-5p | 0.232 | 0.033 | 0.211 | 0.019 | 0.446 |
| hsa-miR-451a | 0.228 | 0.017 | 0.211 | 0.042 | 0.414 |
| hsa-miR-212-3p | 0.454 | 0.080 | 0.240 | -0.055 | 0.962 |
| hsa-miR-143-3p | 0.400 | 0.047 | 0.388 | 0.006 | 0.794 |
| hsa-miR-638 | 0.148 | 0.353 | 0.474 | -0.167 | 0.464 |
| hsa-miR-25-3p | 0.095 | 0.521 | 0.474 | -0.196 | 0.385 |
