# Supplementary material for: Circulating microRNAs and association with methacholine PC20 in the Childhood Asthma Management Program (CAMP) cohort
Source: PLoS One. 2017 Jul 27;12(7):e0180329. doi: 10.1371/journal.pone.0180329 (PMC5531511; doi:10.1371/journal.pone.0180329)
Supplement: S2 Table — (DOCX) [file pone.0180329.s002.docx]

**S2 Table: Circulatory miRNA Association by Least Squares Linear Regression with methacholine PC20 (multivariate model adjusting for age, sex, and height, unranked) with detection of miRNA in at least 50 % of samples**

| **miR** | **miR slope** | **miR p-value** | **FDR p-value** | **95 % CI**  **Lower** | **95 % CI**  **Upper** |
| --- | --- | --- | --- | --- | --- |
| hsa-miR-296-5p | 0.501 | 0.00004 | 0.005 | 0.270 | 0.732 |
| hsa-miR-548b-5p | 0.340 | 0.002 | 0.080 | 0.127 | 0.554 |
| hsa-miR-138-5p | 0.380 | 0.003 | 0.081 | 0.136 | 0.624 |
| hsa-miR-30d-5p | 0.213 | 0.004 | 0.094 | 0.069 | 0.357 |
| hsa-miR-16-5p | 0.206 | 0.004 | 0.094 | 0.066 | 0.347 |
| hsa-miR-1227-3p | 0.323 | 0.006 | 0.120 | 0.094 | 0.552 |
| hsa-miR-203a-3p | 0.206 | 0.007 | 0.128 | 0.056 | 0.356 |
| hsa-miR-128-3p | 0.584 | 0.015 | 0.235 | 0.115 | 1.053 |
| hsa-miR-451a | 0.197 | 0.017 | 0.235 | 0.035 | 0.360 |
| hsa-miR-942-5p | 0.240 | 0.018 | 0.235 | 0.042 | 0.439 |
| hsa-miR-212-3p | 0.291 | 0.022 | 0.267 | 0.042 | 0.540 |
| hsa-miR-143-3p | 0.425 | 0.025 | 0.274 | 0.055 | 0.794 |
| hsa-miR-185-5p | 0.214 | 0.047 | 0.463 | 0.003 | 0.425 |
| hsa-miR-25-3p | 0.224 | 0.048 | 0.463 | 0.002 | 0.445 |
