# Supplementary material for: Circulating microRNAs and association with methacholine PC20 in the Childhood Asthma Management Program (CAMP) cohort
Source: PLoS One. 2017 Jul 27;12(7):e0180329. doi: 10.1371/journal.pone.0180329 (PMC5531511; doi:10.1371/journal.pone.0180329)
Supplement: S3 Table — (DOCX) [file pone.0180329.s003.docx]

**S3 Table: Circulatory miRNA Association by Least Squares Linear Regression with methacholine PC20 (univariate model, ranked) with detection of miRNA in at least 50 % of samples**

| **miR** | **miR slope** | **miR p-value** | **FDR p-value** | **95 % CI Lower** | **95 % CI Upper** |
| --- | --- | --- | --- | --- | --- |
| hsa-miR-296-5p | 0.307 | 0.0003 | 0.021 | 0.142 | 0.473 |
| hsa-miR-138-5p | 0.292 | 0.002 | 0.068 | 0.112 | 0.473 |
| hsa-miR-16-5p | 0.231 | 0.003 | 0.105 | 0.078 | 0.384 |
| hsa-miR-451a | 0.232 | 0.004 | 0.105 | 0.075 | 0.389 |
| hsa-miR-548b-5p | 0.263 | 0.009 | 0.151 | 0.068 | 0.457 |
| hsa-miR-324-3p | 0.285 | 0.009 | 0.151 | 0.072 | 0.499 |
| hsa-miR-942-5p | 0.213 | 0.009 | 0.151 | 0.053 | 0.373 |
| hsa-miR-128-3p | 0.276 | 0.010 | 0.151 | 0.068 | 0.483 |
| hsa-miR-1227-3p | 0.245 | 0.017 | 0.228 | 0.044 | 0.446 |
| hsa-miR-92a-3p | 0.192 | 0.018 | 0.228 | 0.034 | 0.351 |
| hsa-let-7d-5p | 0.202 | 0.028 | 0.330 | 0.023 | 0.381 |
| hsa-miR-30d-5p | 0.170 | 0.033 | 0.363 | 0.014 | 0.326 |
| hsa-miR-145-5p | 0.236 | 0.040 | 0.401 | 0.011 | 0.461 |
| hsa-miR-181c-5p | 0.205 | 0.042 | 0.401 | 0.007 | 0.404 |
| hsa-miR-203a-3p | 0.170 | 0.044 | 0.401 | 0.005 | 0.336 |
