# Supplementary material for: Circulating microRNAs and association with methacholine PC20 in the Childhood Asthma Management Program (CAMP) cohort
Source: PLoS One. 2017 Jul 27;12(7):e0180329. doi: 10.1371/journal.pone.0180329 (PMC5531511; doi:10.1371/journal.pone.0180329)
Supplement: S4 Table — (DOCX) [file pone.0180329.s004.docx]

**S4 Table: Circulatory miRNA Association by Least Squares Linear Regression with methacholine PC20 (multivariate model adjusting for age, sex, and height, ranked) with detection of miRNA in at least 50 % of samples**

| **miR** | **miR slope** | **miR p-value** | **FDR p-value** | **95 % CI Lower** | **95 % CI**  **Upper** |
| --- | --- | --- | --- | --- | --- |
| hsa-miR-296-5p | 0.344 | 0.0001 | 0.016 | 0.173 | 0.515 |
| hsa-miR-138-5p | 0.305 | 0.001 | 0.054 | 0.121 | 0.490 |
| hsa-miR-16-5p | 0.243 | 0.003 | 0.082 | 0.086 | 0.400 |
| hsa-miR-451a | 0.235 | 0.004 | 0.104 | 0.076 | 0.393 |
| hsa-miR-324-3p | 0.330 | 0.005 | 0.110 | 0.103 | 0.557 |
| hsa-miR-548b-5p | 0.281 | 0.007 | 0.136 | 0.079 | 0.484 |
| hsa-miR-942-5p | 0.214 | 0.010 | 0.174 | 0.052 | 0.377 |
| hsa-miR-128-3p | 0.273 | 0.013 | 0.194 | 0.060 | 0.486 |
| hsa-miR-92a-3p | 0.199 | 0.016 | 0.232 | 0.037 | 0.360 |
| hsa-miR-1227-3p | 0.240 | 0.021 | 0.265 | 0.038 | 0.442 |
| hsa-miR-30d-5p | 0.181 | 0.025 | 0.296 | 0.023 | 0.338 |
| hsa-let-7d-5p | 0.198 | 0.033 | 0.364 | 0.016 | 0.379 |
| hsa-miR-145-5p | 0.245 | 0.036 | 0.369 | 0.017 | 0.473 |
| hsa-miR-19b-3p | 0.165 | 0.046 | 0.446 | 0.003 | 0.327 |
