# Supplementary material for: Circulating microRNAs and association with methacholine PC20 in the Childhood Asthma Management Program (CAMP) cohort
Source: PLoS One. 2017 Jul 27;12(7):e0180329. doi: 10.1371/journal.pone.0180329 (PMC5531511; doi:10.1371/journal.pone.0180329)
Supplement: S5 Table — (DOCX) [file pone.0180329.s005.docx]

**S5 Table: miRBase Accession numbers for cytoscape (univariate model, unranked)**

| miR | miRBase Accession # |
| --- | --- |
| hsa-miR-296-5p | MIMAT0000690 |
| hsa-miR-548b-5p | MIMAT0004798 |
| hsa-miR-138-5p | MIMAT0000430 |
| hsa-miR-16-5p | MIMAT0000069 |
| hsa-miR-1227-3p | MIMAT0005580 |
| hsa-miR-203a-3p | MIMAT0000264 |
| hsa-miR-128-3p | MIMAT0000424 |
| hsa-miR-30d-5p | MIMAT0000245 |
