# Supplementary material for: Circulating microRNAs and association with methacholine PC20 in the Childhood Asthma Management Program (CAMP) cohort
Source: PLoS One. 2017 Jul 27;12(7):e0180329. doi: 10.1371/journal.pone.0180329 (PMC5531511; doi:10.1371/journal.pone.0180329)
Supplement: S6 Table — (DOCX) [file pone.0180329.s006.docx]

**S6 Table: DAVID Gene Ontology (GO) Analysis (GOTERM_BP_DIRECT)**

| Term | Number of Genes | Percentage of Genes (%) | P-value | Corrected P-value (Benjamini) |
| --- | --- | --- | --- | --- |
| Translational initiation | 57 | 2.1 | 1.1x10^-13^ | 6.8 x 10^-10^ |
| rRNA processing | 74 | 2.8 | 1.2 x 10^-12^ | 3.6 x 10^-9^ |
| Protein stabilization | 54 | 2.0 | 1.7 x 10^-12^ | 3.5 x 10^-9^ |
| Positive regulation of transcription, DNA-templated | 131 | 4.9 | 9.9 x 10^-11^ | 1.5 x 10^-7^ |
| Negative regulation of apoptotic process | 118 | 4.4 | 1.1 x 10^-9^ | 1.3 x 10^-6^ |
